# B-cell tolerance and autoimmunity

**DOI:** 10.12688/f1000research.10583.1

**Published:** 2017-03-29

**Authors:** Takeshi Tsubata

**Affiliations:** 1 Department of Immunology, Medical Research Institute, Tokyo Medical and Dental University, Tokyo, 113-8510, Japan

**Keywords:** b cell, b cell tolerance, autoimmunity, self-reactive b cells

## Abstract

Self-reactive B cells are tolerized at various stages of B-cell development and differentiation, including the immature B-cell stage (central tolerance) and the germinal center (GC) B-cell stage, and B-cell tolerance involves various mechanisms such as deletion, anergy, and receptor editing. Self-reactive B cells generated by random immunoglobulin variable gene rearrangements are tolerized by central tolerance and anergy in the periphery, and these processes involve apoptosis regulated by Bim, a pro-apoptotic member of the Bcl-2 family, and regulation of B-cell signaling by various phosphatases, including SHIP-1 and SHP-1. Self-reactive B cells generated by somatic mutations during GC reaction are also eliminated. Fas is not directly involved in this process but prevents persistence of GC reaction that allows generation of less stringently regulated B cells, including self-reactive B cells. Defects in self-tolerance preferentially cause lupus-like disease with production of anti-nuclear antibodies, probably due to the presence of a large potential B-cell repertoire reactive to nucleic acids and the presence of nucleic acid-induced activation mechanisms in various immune cells, including B cells and dendritic cells. A feed-forward loop composed of anti-nuclear antibodies produced by B cells and type 1 interferons secreted from nucleic acid-activated dendritic cells plays a crucial role in the development of systemic lupus erythematosus.

## Introduction

Studies on autoantibody-transgenic mice and analyses of the repertoire of various B-cell subsets in humans and mice have demonstrated that self-reactive B cells are negatively regulated at various stages of B-cell development and maturation, including the immature B-cell stage in bone marrow, transitional B-cell stage, and germinal center (GC) B-cell stage ^1, 2^. There are multiple mechanisms for B-cell tolerance, such as deletion, functional inactivation (anergy), and alteration of antigen specificity by replacement of immunoglobulin (Ig) variable (V) gene segments (receptor editing) ^3, 4^. Some self-reactive B cells emerge in the peripheral lymphoid organs without being deleted or functionally inactivated but do not differentiate to plasma cells even in the presence of interaction with self-antigens as if they are ignored (clonal ignorance) ^2^. In some autoantibody-transgenic mice, self-reactive B cells are accumulated in marginal zone B cells ^5– 7^, suggesting that these self-reactive B cells are positively selected to differentiate to marginal zone B cells. However, self-reactive marginal zone B cells are also tolerized ^8^.

Autoantibodies to nuclear antigens are characteristically produced in patients with systemic lupus erythematosus (SLE) and its animal models and play a pathogenic role in the development of this disease. Production of these autoantibodies requires a break of B-cell tolerance because B cells reactive to nuclear antigens have been shown to be tolerized ^1, 9^. Genes expressed in B cells are enriched in SLE-associated genes whereas those expressed in CD4 ^+^ T cells are enriched in genes associated with rheumatoid arthritis ^10^, suggesting that defects in B cells play a central role in the development of SLE. In mice, SLE-like disease is the most common autoimmune disease developed by genetic defects in B cells ^11^. These defects include those that break B-cell tolerance by regulating B-cell activation and survival irrespective of antigen specificity ^12, 13^. Thus, general (antigen-non-specific) defects in B-cell tolerance induce production of autoantibodies to nuclear antigens, suggesting the presence of mechanisms for preferential production of these autoantibodies. One of the mechanisms appears to be a large potential B-cell repertoire reactive to nuclear antigens ^1^. In both humans and mice, reactivity to nuclear antigens is demonstrated in more than half of immature B cells in which the B-cell repertoire is formed by random recombination of Ig V gene segments but not yet selected by antigens. Another mechanism involves nucleic acid (NA) sensors that activate various cell types, including B cells, upon interaction with NAs ^14– 16^. NA sensors play a crucial role in the defense against microbes, especially viruses, through recognition of microbial DNA and RNA but also are involved in the activation of B cells reactive to nuclear antigens containing NAs, leading to production of autoantibodies to nuclear antigens. Thus, development of SLE involves both functional defects in B cells and NA-induced activation of immune cells. Crucial roles of these mechanisms are supported by the findings that SLE-associated genes in humans contain a number of genes involved in the regulation of B-cell signaling, NA degradation, or sensing of NAs, including the NA sensors TLR7 and TLR9 ^17, 18^. In this review, I discuss mechanisms for B-cell tolerance and its break in SLE with a focus on regulation of B-cell signaling and NA-mediated immune cell activation. I also discuss tolerance of self-reactive B cells generated by somatic mutations of Ig V genes in GC reaction and the contribution of GC reaction in autoimmunity.

## Nucleic acid-induced B-cell activation and interferons

**Figure 1.**
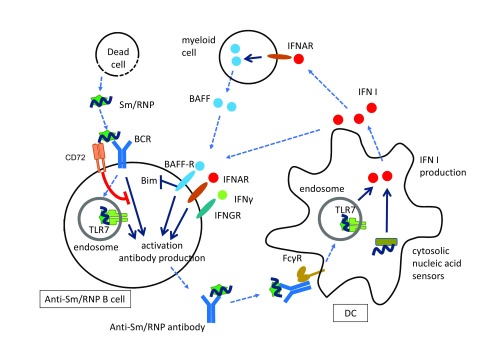
A feed-forward loop consisting of anti-Sm/RNP antibody and type 1 interferons (IFN I) and Sm/RNP-specific B-cell inhibition by CD72. The nuclear self-antigen Sm/RNP released from dead cells is recognized by Sm/RNP-reactive B cells and generates both B-cell antigen receptor (BCR) signaling and co-stimulatory signaling through TLR7 in the endosome. The combination of these two signaling pathways induces cell activation and production of anti-Sm/RNP antibody. The immune complex consisting of Sm/RNP and anti-Sm/RNP antibody is endocytosed by dendritic cells (DCs) through interaction with Fcγ receptor and is recognized by TLR7 in endosome, resulting in the production of IFN I. IFN I is also produced through recognition of nucleic acids (NAs) by cytosolic NA sensors. IFN I activates B cells through receptor for IFN I (IFNAR) to induce IFN-inducible genes, including TLRs. IFN I also activates B cells indirectly by inducing B cell-activating factor (BAFF) expression in myeloid cells. BAFF inhibits expression of Bim and perturbs B-cell tolerance. IFNγ is also involved in the activation of self-reactive B cells. CD72 recognizes Sm/RNP and specifically inhibits BCR signaling when BCR recognizes Sm/RNP, thereby inhibiting production of anti-Sm/RNP antibody. BAFF-R, B cell-activating factor receptor.

TLR7 and TLR9 are endosome-localizing innate NA sensors recognizing RNA and DNA, respectively, and are involved in immune responses to microbes, especially viruses, by recognizing microbial NAs ^14^. They are also involved in autoantibody production to NA-related self-antigens ^15, 16^. Patients with SLE produce autoantibodies to the complexes of NAs and nuclear proteins such as nucleosomes and Sm/RNP. Nucleosomes and Sm/RNP contain DNA and RNA, respectively, and thus are recognized by TLR9 and TLR7, respectively ^19, 20^, both of which are expressed in B cells as well as innate immune cells. When these nuclear self-antigens are released from dead cells, they interact with B cells reactive to these self-antigens through B-cell antigen receptor (BCR) and are translocated together with BCR to endosomes where they are recognized by TLR7 and TLR9 ( Figure 1). In these self-reactive B cells, the combination of signaling through BCR and co-stimulatory signaling through TLRs induces cell activation, leading to production of autoantibodies to the nuclear self-antigens. Complexes of NAs and nuclear proteins are more immunogenic than NAs alone and this is probably due to resistance to degradation by nucleases. Deficiency in TLR7 and TLR9 markedly reduces autoantibody to Sm/RNP and DNA, respectively, in lupus-prone mice ^21^, clearly indicating that B-cell activation mediated by NA sensors facilitates production of autoantibodies to nuclear antigens. Although both TLR7 and TLR9 are involved in autoantibody production to nuclear self-antigens, TLR7 but not TLR9 is required for the development of autoimmune disease in a mouse model ^21^. TLR9 rather ameliorates TLR7-dependent development of lupus-like disease ^22, 23^ by competing endosomal transport with TLR7 ^24^. On the basis of these findings, together with the genetic findings, demonstrating the association of RNA-sensing pathways with lupus, a dominant role of RNA-related antigens in the development of lupus is suggested ^16^, although the mechanism is not yet understood.

In leukocytes from patients with SLE, expression of genes responsive to type 1 interferons (IFN I) is markedly augmented ^25, 26^ and this is probably due to augmented IFN I production. The augmented IFN I production in SLE appears to involve TLR7 and TLR9 as signaling through these TLRs induces IFN I expression in innate immune cells ^27^, especially in plasmacytoid dendritic cells (DCs), which are capable of producing IFN I in large quantity. Upon forming immune complexes with autoantibodies, nuclear self-antigens, such as Sm/RNP ^28^, and DNA complexed with HMGB1 ^29^ are endocytosed by FcγR in DCs and recognized by TLR7 and TLR9 in endosomes, leading to the production of a large amount of IFN I ( Figure 1). Some of the patients with type I interferonopathies, a set of Mendelian disorders such as Aicardi-Goutières syndrome (AGS) characterized by constitutive IFN I production, and their animal models develop lupus-like autoimmune disease as well as various inflammatory lesions ^30^. These observations suggest that IFN I perturbs self-tolerance to nuclear self-antigens and induces the development of lupus. Involvement of IFN I in development of lupus is further supported by the finding on a pristane-induced lupus model. Although most of the lupus-prone mice do not show a strong IFN signature, the pristane-induced lupus model shows a strong TLR7-dependent IFN signature ^31^ and requires the receptor for IFN I (IFNAR) for both autoantibody production and development of lupus ^32^. IFN I activates the expression of a large number of genes in various immune cells, including genes involved in B-cell activation ^33^ such as TLRs in B cells ^34^ and genes for B cell-activating molecules such as B cell-activating factor (BAFF) ^35^ ( Figure 1). The products of IFN I-inducible genes may collectively abrogate self-tolerance and activate self-reactive B cells. The majority of mutations found in AGS are located in genes involved in the metabolism of cytosolic NAs and their recognition ^30^. Some of these genes such as *TREX1* ^36^ encoding a cytosolic nuclease and *IFIH1* ^37^ encoding the cytosolic RNA sensor MDA5 are associated with SLE in humans. Thus, IFN I production caused by augmented responses to cytosolic NAs appears to be involved in SLE as well as AGS. Taken together, autoantibodies to nuclear antigens complexed with self-antigens induce IFN I production in DCs, and IFN I induces production of the autoantibodies in B cells, resulting in a feed-forward loop ( Figure 1). This feed-forward loop may cause a strong IFN signature and massive production of autoantibodies to nuclear self-antigens characteristic of SLE.

Although the pristane-induced lupus model shows a type I IFN signature and requires IFN I for autoantibody production and disease development, IFNγ is overproduced and plays a crucial role in various other mouse models such as MLR-Faslpr/lpr, Sle1b, and Wiskott-Aldrich syndrome (WAS) chimera mice ^31, 38–40^. In both WAS chimera and Sle1b mice, B cell-specific deletion of IFNγ receptor abrogates spontaneous GC formation, autoantibody production, and the development of lupus-like disease ^39, 40^, suggesting a crucial role of IFNγ in breach of B-cell tolerance. In spite of the considerable overlap between genes induced by IFN I and those induced by IFNγ, IFNγ-induced gene expression is evident in patients with SLE because these patients overexpress the IFNγ-inducible genes whose expression is genes suppressed by *in vivo* IFNγ blockade ^41^. Thus, IFNγ as well as IFN I may play a role in the pathogenesis of human SLE as well as mouse models.

## Regulation of central tolerance and clonal anergy by apoptosis and phosphatases

Self-reactive B cells generated in bone marrow by random Ig V gene rearrangements are tolerized by central tolerance such as deletion, anergy and receptor editing. It is already established that Bim, a pro-apoptotic member of the Bcl-2 family, plays a crucial role in the deletion and anergy of self-reactive B cells generated in bone marrow by regulating apoptosis ^42– 44^. Self-reactive B cells in Bim ^−/−^ autoantibody-transgenic mice clearly escape from both deletion and anergy ^42, 43^. Bim is required for BCR ligation-induced B-cell apoptosis that appears to be involved in the deletion of self-reactive B cells ^42^. Bim is also involved in premature death of anergic B cells as they are less sensitive to survival signaling generated by BAFF ^43^ that induces B-cell survival by reducing Bim expression ^45^. Thus, Bim-mediated apoptosis plays a crucial role in both the deletion and anergy of self-reactive B cells. Breach of deletion and anergy in self-reactive Bim ^−/−^ B cells may contribute to the development of lupus-like disease in Bim ^−/−^ mice ^46^.

The lipid phosphatase SHIP-1 and the non-receptor type protein tyrosine phosphatases (PTPs) SHP-1 and LYB/PEP regulate B-cell tolerance and the development of autoimmune diseases ^47, 48^. A recent study by Getahun *et al*. ^48^ demonstrated that inducible deletion of either SHP-1 or SHIP-1 reverses anergy of DNA-reactive B cells and allows spontaneous differentiation of these self-reactive B cells to plasma cells. This result clearly indicates that anergy of self-reactive B cells is reversible and that both SHP-1 and SHIP-1 are required for maintenance of anergy. B cell-specific deletion of SHP-1 or SHIP-1 causes severe lupus-like disease with autoantibody production ^12, 13^, suggesting that a functional defect in B cells caused by deletion of SHP-1 or SHIP-1 is sufficient to abrogate B-cell tolerance and to develop autoimmune disease.

**Figure 2.**
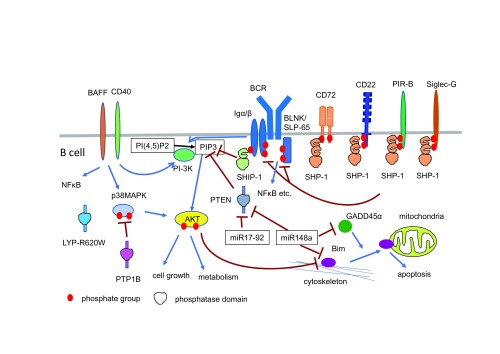
B-cell signaling pathways regulating B-cell tolerance and autoimmunity involve phosphatases and microRNAs. Phosphatases such as SHP-1, SHIP-1, and LYP-R620W (and also mouse ortholog PEP-R619W) and microRNAs such as miR148 and miR17-92 are demonstrated to reverse central B-cell tolerance, B-cell anergy or both. PTP1B, a protein tyrosine phosphatase, and phosphatase and tensin homolog (PTEN), a lipid phosphatase, are also involved in B-cell tolerance as B cell-specific deletion of these genes causes lupus-like disease ^56, 88^. SHIP-1 is activated by mono-phosphorylated immuno-receptor tyrosine-based activation motif at Igα/β. Both SHIP-1 and PTEN dephosphorylate PIP3 required for activation of AKT involved in cell activation and survival. SHP-1 is activated by phosphorylated immuno-receptor tyrosine-based inhibition motifs at various inhibitory co-receptors such as CD72, CD22, PIR-B, and Siglec-G and dephosphorylates proximal B-cell antigen receptor (BCR) signaling molecules such as Igα/β and BLNK/SLP-65, thereby reducing BCR signaling, including AKT activation. PTP1B dephosphorylates p38MAPK and negatively regulates AKT activation induced by signaling through CD40 and B cell-activating factor (BAFF) receptor. LYP-R620W and its mouse ortholog PEP-R619W perturb B-cell tolerance. The microRNA miR148a inhibits expression of Bim, GADD45α, involved in Bim translocation to mitochondria, and PTEN, thereby suppressing Bim-mediated B-cell deletion and augmenting AKT activation. PTEN expression is also suppressed by miR17-92. NF-κB, nuclear factor-kappa B; PI-3K, phosphatidyl inositol 3-kinase; PI(4,5)P2, phosphatidylinositol 4,5-bisphosphate; PTP, protein tyrosine phosphatase.

In B cells, both SHP-1 and SHIP-1 negatively regulate signaling through BCR. SHIP-1 dephosphorylates phosphatidyl inositol 3,4,5-triphosphate (PI(3,4,5)P3), required for phosphatidyl inositol 3-kinase (PI-3K)-mediated activation of AKT, which in turn activates various signaling molecules, including mechanistic target of rapamycin (mTOR), and regulates cell activation processes, including metabolism, proliferation, and cytoskeletal changes ^49^. The PI-3K pathway as well as the nuclear factor-kappa B (NF-κB) pathway plays a crucial role in BCR and BAFF-R signaling for B-cell survival and activation ^50, 51^. Thus, SHIP-1 inhibits B-cell survival and activation by negatively regulating the PI-3K pathway. SHP-1 dephosphorylates proximal BCR signaling molecules such as Igα/Igβ and SLP-65/BLNK ^52^ required for BCR signaling, including the PI-3K pathway. Both SHP-1 and SHIP-1 contain SH2 domains, and their activation requires binding of these SH2 domains to tyrosine-phosphorylated proteins. When BCR interacts with antigens, BCR-associated tyrosine kinases such as Syk and Lyn phosphorylate various cytoplasmic signaling molecules ^53^. Lyn also phosphorylates B-cell co-receptors, including CD19, CD22, PIR-B, and CD72. Upon phosphorylation, CD19 recruits and activates PI-3K. In contrast, other co-receptors such as CD22, PIR-B, and CD72 recruit SHP-1 at the phosphorylated immuno-receptor tyrosine-based inhibition motifs (ITIMs) in their cytoplasmic regions and activate SHP-1 ^54^ ( Figure 2). Although fully phosphorylated immuno-receptor tyrosine-based activation motifs (ITAMs) in Igα/Igβ recruit the tyrosine kinase Syk, these ITAMs are partially phosphorylated in anergic self-reactive B cells. The partially phosphorylated ITAMs recruit and activate SHIP-1 instead of Syk ^47^. Probably owing to continuous interaction of BCR with self-antigens in self-reactive B cells, both SHP-1 and SHIP-1 are constitutively activated in anergic B cells and play a crucial role in the maintenance of anergy by suppressing the PI-3K/AKT pathway.

PTP1B is another non-receptor type PTP known to regulate metabolic signaling pathway ^55^. B cell-specific PTP1B deficiency causes augmented B-cell responses to BAFF, CD40 ligation, and lipopolysaccharide but not BCR ligation and induces lupus-like disease with autoantibody production ^56^. PTP1B therefore appears to regulate B-cell tolerance as is the case for SHP-1, although PTP1B regulates a signaling pathway distinct from those regulated by SHP-1 ( Figure 2). PTP1B dephosphorylates p38 MAPK as a direct substrate and regulates AKT.

The crucial role of both apoptosis and regulation of the PI-3K pathway in B-cell tolerance is also demonstrated in the studies to isolate microRNAs that inhibit deletion of self-reactive B cells at the immature and mature B-cell stage ^57, 58^. These studies demonstrated that expression of miR-148a or the miR17-92 family reverses deletion of self-reactive B cells. miR-148a protects B cells from deletion by suppressing Gadd45α, phosphatase and tensin homolog (PTEN), and Bim ( Figure 2). PTEN is a lipid phosphatase that regulates the PI-3K pathway by catalyzing PI(3,4,5)P3. Gadd45α was reported to induce translocation of Bim to mitochondria where Bim inhibits apoptosis ^59^, and its defect causes lupus-like disease ^60^. miR17-92 also regulates PTEN ^57, 61^. Thus, Bim and the PI-3K pathway regulated by PTEN play a crucial role in microRNA-mediated regulation of B-cell tolerance.

PEP is a mouse ortholog of human LYP encoded by PTPN22. The PTPN22-C1858T haplotype that encodes LYP-R620W is associated with various autoimmune diseases, including SLE, Graves’ disease, type 1 diabetes, and rheumatoid arthritis ^17, 18^. PEP/LYP binds to Csk, but this association is disrupted in LYP-R620W ^62^. Although PEP-deficient mice do not show much phenotype in B cells, mice expressing PEP-R619W carrying the corresponding mutation with human LYP-R620W show hyperactivity of both B and T cells and development of lupus-like disease ^63, 64^, suggesting that functional properties of LYP acquired by the R620W mutation cause B-cell hyperactivity. In humans, both transitional and mature naïve B cells from individuals with LYP-R620W show much higher frequencies in self-reactive B cells ^65^, indicating defects in central B-cell tolerance. This defect is corrected by enzymatic inhibitor of LYP ^66^. Thus, either augmented phosphatase activity or altered functional property due to lack of Csk binding in LYP-R620W may perturb central B-cell tolerance, although its substrates regulating tolerance are not yet clear.

## Nuclear antigen-specific tolerance mechanism

Among various B-cell inhibitory co-receptors that activate SHP-1, CD72 plays a unique role in specifically tolerizing B cells reactive to nuclear antigens ^67^. Recently, Akatsu *et al*. demonstrated that CD72 specifically binds to Sm/RNP and that CD72-mediated signal inhibition is induced when B cells interact with Sm/RNP through BCR, leading to inhibition of B-cell responses to Sm/RNP ( Figure 1) ^67^. Sm/RNP may co-ligate Sm/RNP-reactive BCR and CD72, thereby inducing phosphorylation of the CD72 ITIM by Lyn associated with BCR, the event required for SHP-1 activation and signal suppression ^54^. This finding is consistent with the previous findings that CD72-deficient mice develop lupus-like disease much more severely than mice deficient in other inhibitory receptors such as CD22 and PIR-B ^68, 69^, although CD72 does not regulate polyclonal BCR signaling induced by anti-IgM antibody ^70^. By specifically suppressing signaling through BCR reactive to nuclear antigens, CD72 strongly inhibits the development of lupus without affecting polyclonal BCR signaling.

NA sensors, including TLR7, respond to microbial RNA better than endogenous RNA by recognizing the structural features of microbial RNA such as dsRNA and 5′-triphosphate RNA as well as features such as localization of RNA ^71, 72^. Nonetheless, TLR7 plays a crucial role in autoimmune responses by recognizing the RNA-containing self-antigen Sm/RNP ^20, 21^. Thus, mechanisms intrinsic to TLR7 may not completely distinguish self-RNA from microbial RNA and CD72 is required for complete suppression of responses to self-RNA. CD72 appears to recognize RNA-related self-antigens but not microbial RNA ^67^. Microbial RNA is thus distinguished from self-RNA by both mechanisms intrinsic in NA sensors and specific recognition of NA-containing self-antigen by CD72.

B-1 cells are suggested to play a role in autoimmune diseases because (1) self-reactive and poly-reactive B cells are positively selected and accumulated in B-1 cells and (2) B-1 cell expansion is associated with autoimmune diseases in both humans and mice ^73^. Indeed, B-cell SHP-1 regulates both development of lupus-like disease and B-1 cell expansion ^12^. However, CD72 regulates the former but not the latter. In contrast, mice deficient in Siglec-10, a SHP-1-recruiting inhibitory receptor abundantly expressed in B-1 cells, show marked expansion of B-1 cells ^74^ but development of only mild disease in aged mice older than 1 year of age ^68^. Thus, SHP-1 regulates B-1 cell expansion and development of lupus-like disease through distinct SHP-1-recruiting receptors, Siglec-G and CD72, respectively, and B-1 cell expansion does not necessarily associate with development of autoimmune disease.

## Tolerance of germinal center B cells and maturation of self-reactive B cells to plasma cells

Antigen-stimulated B cells differentiate to plasma cells either directly by extrafollicular pathway or through GC reaction, in which B cells undergo Ig diversification by somatic hypermutation in the Ig V region and are selected for production of high-affinity antibody. It is established that somatic mutations of Ig V genes play a role in the generation of self-reactive B cells ^75, 76^. Comparison of the sequence of autoantibodies and their germline genes demonstrated that many of the autoantibodies are generated from non-self-reactive antibodies and acquire self-reactivity by somatic mutations in Ig V regions, although some autoantibodies are derived from germline-encoded autoantibodies.

Involvement of GC reaction in autoantibody production in lupus is further supported by indirect evidence. First, mice that spontaneously develop lupus show spontaneous GC reaction ^77^, although immunization is required for GC formation in normal mice. Second, GC B cells strongly express Fas, a member of tumor necrosis factor receptor family transmitting apoptotic signaling. B cell-specific deletion of Fas induces the development of lupus-like disease ^78^, suggesting that Fas-mediated apoptosis of self-reactive GC B cells is involved in self-tolerance for nuclear antigens.

The presence of self-tolerance that tolerizes self-reactive GC B cells generated by somatic mutations was clearly demonstrated by using transgenic mice for anti-hen egg lysozyme (HEL) antibody and mice transgenic for mutated HEL as a surrogate self-antigen. In this elegant experimental system, the mutated HEL is recognized by somatically mutated anti-HEL antibody generated upon affinity maturation but not by un-mutated anti-HEL antibody. Therefore, B cells reactive to the mutated HEL represent self-reactive B cells generated by somatic mutations. This study demonstrated that self-reactive B cells generated by somatic mutations are efficiently eliminated if the reactive self-antigens are present within GCs ^79, 80^. In the absence of Fas, self-reactive B cells generated by somatic mutations are efficiently eliminated, but GC reaction persists for a prolonged period and generates “rogue GC B cells” that accumulate somatic mutations but are not stringently selected. The “rogue B cells” show a defect in affinity maturation and gain self-reactivity ^81^. Thus, Fas is not directly required for elimination of self-reactive B cells generated by somatic mutations but is required for the prevention of prolonged GC reaction that generates “rogue GC B cells” due to less stringent selection, thereby indirectly inhibiting generation of self-reactive GC B cells.

Although evidence suggests the involvement of GC reaction in the generation of self-reactive B cells, studies using rheumatoid factor-transgenic mice demonstrated that self-reactive B cells are excluded from GCs and differentiate to plasma cells by the extrafollicular pathway accompanied with somatic mutations of Ig V genes ^82, 83^. The crucial role of the extrafollicular pathway in autoantibody production is also supported by the deep sequencing analysis of Ig V genes in B cells from patients with SLE ^84^. This analysis revealed that the Ig V regions of plasma blasts from patients with SLE contain fewer somatic mutations compared with those generated by vaccination and are similar in sequence to those of recently activated B cells but not memory B cells. Thus, involvement of GC reaction and defect in the GC checkpoint in autoantibody production in autoimmune diseases need to be further addressed in the future.

## Conclusions

Self-reactive B cells are tolerized by multiple different mechanisms at multiple different B-cell differentiation stages. Autoantibody production therefore requires self-reactive B cells to survive multiple selections at different B-cell differentiation stages. This explains the synergy of genetic defects such as deficiency of Fas and Bim ^85– 87^ and deficiency of CD72 and Fas ^70^ in the development of severe autoimmune disease. Nonetheless, various single-gene defects cause autoantibody production and autoimmune diseases. At least some of these genes may breach only a part of the checkpoints, but their defect is sufficient for autoantibody production probably because each checkpoint is not able to completely deplete self-reactive B cells and may become less stringent by aging and environmental factors. Moreover, the presence of multiple mechanisms suggests multiple pathways for autoantibody production, including GC and extrafollicular pathways. Further studies will reveal more precise mechanisms by which self-reactive B cells are generated and differentiate to autoantibody-producing cells in autoimmune disease.
